# Student Motivation Analysis Based on Raising-Hand Videos

**DOI:** 10.3390/s24144632

**Published:** 2024-07-17

**Authors:** Jiejun Chen, Miao Wang, Liang Wang, Fuquan Huang

**Affiliations:** 1 School of Electronics and Communications Engineering, Sun Yat-sen University, Shenzhen 518107, China; chenjj289@mail2.sysu.edu.cn; 2 School of Education, South China Normal University, Guangzhou 510898, China; wmm1903@163.com

**Keywords:** smart education, computer vision, intelligent perception, motion behavior analysis, morphological analysis

## Abstract

In current smart classroom research, numerous studies focus on recognizing hand-raising, but few analyze the movements to interpret students’ intentions. This limitation hinders teachers from utilizing this information to enhance the effectiveness of smart classroom teaching. Assistive teaching methods, including robotic and artificial intelligence teaching, require smart classroom systems to both recognize and thoroughly analyze hand-raising movements. This detailed analysis enables systems to provide targeted guidance based on students’ hand-raising behavior. This study proposes a morphology-based analysis method to innovatively convert students’ skeleton key point data into several one-dimensional time series. By analyzing these time series, this method offers a more detailed analysis of student hand-raising behavior, addressing the limitations of deep learning methods that cannot compare classroom hand-raising enthusiasm or establish a detailed database of such behavior. This method primarily utilizes a neural network to obtain students’ skeleton estimation results, which are then converted into time series of several variables using the morphology-based analysis method. The YOLOX and HrNet models were employed to obtain the skeleton estimation results; YOLOX is an object detection model, while HrNet is a skeleton estimation model. This method successfully recognizes hand-raising actions and provides a detailed analysis of their speed and amplitude, effectively supplementing the coarse recognition capabilities of neural networks. The effectiveness of this method has been validated through experiments.

## 1. Introduction

Research on intelligent education has been a hot topic; in the 1990s, the US government announced the widespread use of the internet in educational architecture design and vigorously developed the distance education industry [1]. The MIT Laboratory in the United States was the first to apply affective computing technology to the field of online teaching. In 2005, the researchers studied specific teaching systems and used sensors that came into contact with the human body to assist in obtaining emotional information [2]. Some researchers are dedicated to computing human emotional states [3]. Many European countries have also conducted a lot of research on facial recognition technology and emotional computing, such as many sensor-based wearable computers [4]. Many emotional research groups have been formed in Europe, and some of the more famous ones include the Free University of Brussels’ emotional research on intelligent interactive robots at the end of the twentieth century [5]. Furthermore, Damasin found that emotions, rational thinking, and logical reasoning abilities complement each other, and emotional ability plays an important role in human perception, planning, reasoning, learning, memory, decision-making, and creativity [6]. Some researchers have defined classroom attention [7,8,9], while others have been dedicated to using various emotional sensors to obtain emotional signals [10].

Many schools have already used information and communication technology [11,12,13] to build smart classrooms to help teachers improve students’ learning effectiveness. One commonly used tool in smart classrooms is video monitoring systems [14,15]. The video monitoring system in the smart classroom can record student performances and interactions in the classroom, such as their concentration, participation, cooperation, etc., so that teachers can better understand students’ learning status and needs.

The data format of student classroom performance recorded by the video monitoring system in the smart classroom mainly includes: video recordings (the video monitoring system in the smart classroom saves classroom recordings, which include student performances and interactions in the classroom. Teachers can better understand student performances and interactions in the classroom by watching the recordings), and student information (the video monitoring system in the smart classroom also records students’ basic information, such as their names, grades, classes, etc. This information can help teachers provide personalized teaching and management for students), classroom data analysis (the video monitoring system in the smart classroom can record student performances and interactions in the classroom by analyzing the recordings, such as their participation, accuracy in answering questions, and interactions with classmates. The data can help teachers better understand the learning situations and needs of students, and develop more scientific teaching plans), and teaching feedback (the video monitoring system in the smart classroom can also provide teaching feedback. For example, teachers can provide students or their parents with feedback on their performance in the classroom. This can help students better understand their own learning situations and make targeted improvements to their learning methods and attitudes).

Many studies have implemented the detection of hand-raising actions in the classroom based on the information provided by video monitoring systems [16,17,18,19]. For example, Si et al. [16] and Le et al. [17] proposed a method based on the RCNN network of deep learning to detect hand-raising actions in the classroom. Lin et al. [18] proposed a deep learning method based on the RFCN network to detect hand-raising actions in a real classroom environment. Liao et al. [19] proposed a two-stage method to recognize hand-raising gestures in a real classroom scenario. In the first stage, multi-stage posture estimation is performed to obtain the candidate hand regions of each student. Key points are assembled only on the raised arm and multi-scale information fusion is used to obtain more accurate results. In the second stage, the gesture style is recognized by a binary classification network.

However, there are some key challenges in the current research area. The first involves insufficient technical infrastructure: While smart classrooms and intelligent education hold great theoretical potential, many schools and educational institutions lack adequate technical infrastructure for practical application. Issues such as insufficient broadband network coverage and slow device updates limit the widespread adoption of intelligent education. The second involves inadequate teacher training and support: Implementing smart classrooms requires teachers to possess certain technical knowledge and operational skills. However, there is still significant room for improvement in terms of teacher training and support. Many teachers have low acceptance and proficiency in using new technologies, resulting in sub-optimal use of intelligent education tools and platforms. The third involves data privacy and security: Intelligent education relies heavily on personal data from students and teachers, raising concerns about data privacy and security. Balancing educational quality with protecting user privacy and data security is a critical challenge in intelligent education. The fourth involves innovation in products and services: The rapid development of the intelligent education field necessitates continuous innovation and optimization of products and services. For instance, intelligent educational robots as new teaching tools require constant improvement in their product form and performance. Additionally, they require accompanying professional course content and service support to enhance their effectiveness and educational value.

**Figure 1:**
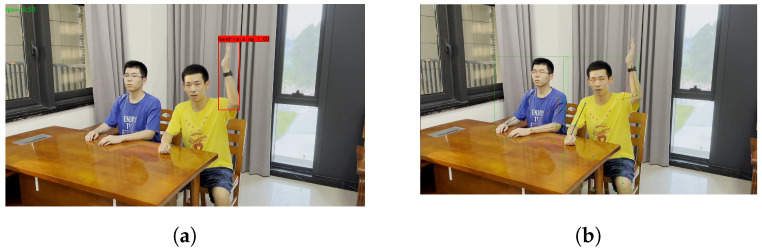
These images are the visualization results of two different methods. (**a**) Merely hand-raising recognition. (**b**) With skeleton information.

By analyzing and summarizing the above relevant research results, it is easy to find that existing research methods have some limitations, such as the inability to compare the enthusiasm of raising hands in the classroom and the inability to establish a detailed database of the hand-raising behaviors of students after class. This paper aims to provide more detailed information about student behaviors in class, especially hand-raising behaviors, which the above studies cannot achieve. The morphological-based method proposed in this paper supplements the existing methods by allowing real-time analysis of students’ hand-raising behavior in the classroom and the establishment of a detailed database of the hand-raising behaviors of students, which provides strong support for students’ learning effectiveness. For example, in Figure 1, the left subfigure shows an example of a hand-raising recognition application in a classroom, which only recognizes the hand-raising action without analyzing the enthusiasm of the person raising their hand. In the right subfigure, we can see that a polygon can be formed by a single arm. A student has two arms, and the polygonal area is bounded by line segments. Using skeleton estimation and morphological calculation, the key points in the student’s skeleton estimation results are shown in the figure. The area surrounded by the lines connected by blue and red represents the region considered in the study, and the angles in purple indicate the angle information of interest.

**Figure 2:**
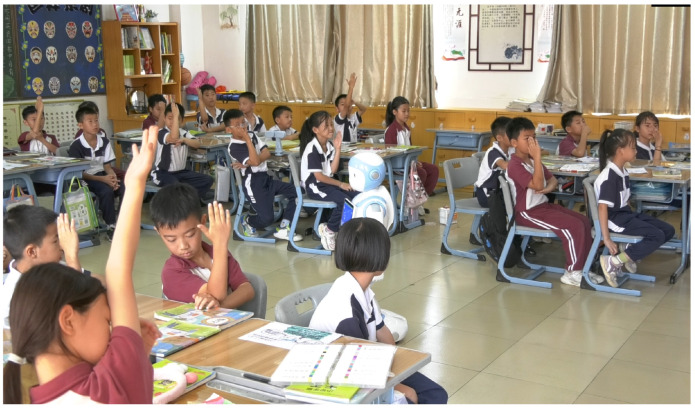
This is an example of the multi-support intelligent classroom, where AI robots are competent in all subjects offered by the school and serve as the teacher, while human teachers assist in teaching.

Figure 2 is an example of a researcher-developed robot used in a smart classroom, in which the robot can autonomously teach a course, walk around the classroom, ask students questions, and provide feedback. If a more detailed analysis of student behavior in the classroom can be achieved, the robot in the face of such questions as multiple students raise their hands to choose which student to answer the question, can independently make the best choice, not only to drive the classroom motivation, but also in the follow-up of the classroom performance of different students for personalized tracking and guidance. With the activity analysis method proposed in this paper, teachers and robot teachers can gain a deeper understanding of student behaviors and can decide which student to choose to answer the question to achieve better teaching results in classrooms. And this explains the motivation of this paper.

**Figure 3:**
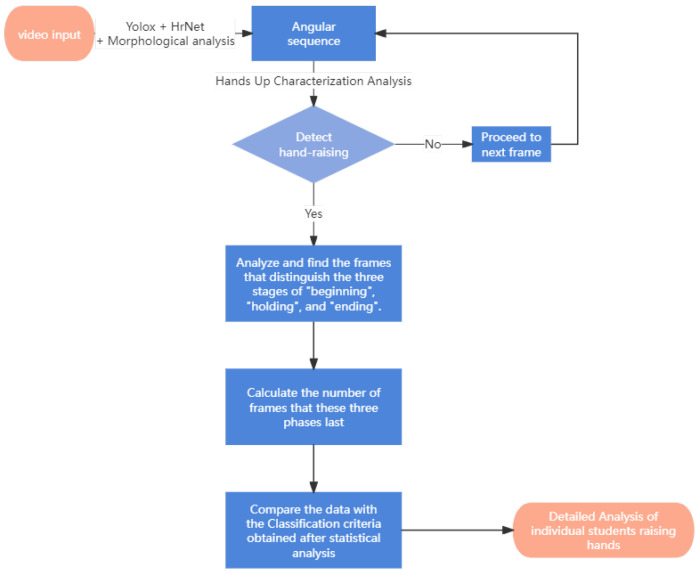
Angular analysis flowchart.

To better study the characteristics of hand-raising with different amplitudes and speeds, the data used in this study consisted of videos specifically recorded by researchers. These included videos of single-person, single-time hand-raising; single-person, multiple-time hand-raising; multiple-person, single-time hand-raising; and multiple-person, simultaneous hand-raising. These videos are equivalent to recording information in a video monitoring system. The method used in this experiment first uses the deep learning models YOLOX [20,21] and HrNet [22] to obtain the skeleton estimation results of students. Based on the skeleton estimation results, the morphological analysis method is used to calculate the key measurements (such as the area formed by the arm joint and the angle formed by it) that can describe the hand-raising action of the student in the current frame. The hand-raising action is successfully recognized and the complete hand-raising action is divided into three stages: “beginning”, “holding”, and “ending”. The speed and amplitude of raising hands are analyzed in more detail, achieving the task of detailed classification of hand-raising action. This is crucial for smart classrooms because it not only allows comparison of the enthusiasm of raising hands among multiple students raising hands simultaneously but also provides structured data (the number of times a student raises his/her hand and the corresponding speed and amplitude, i.e., enthusiasm information) that can be saved for subsequent analysis and teacher intervention after class. The main highlight of this article is to address the problem of “how to choose a more suitable student from a large number of students who raise their hands to answer the teacher” by proposing a method that converts student skeleton key point information into several one-dimensional time series using morphology-based analysis and analyzes them to achieve classification and recognition of different hand-raising amplitudes and speeds. Figure 3 shows a detailed angular analysis flowchart, in which angular sequence is first extracted by using Yolox and HrNet network, after morphological analysis. Then the sequence goes to the next process of detecting hand-raising behavior. If such behavior is detected, the sequence is analyzed to obtain the frames that distinguish the three stages of “beginning”, “holding”, and “ending”. After that, the duration of these three phases can be calculated in terms of the number of frames. These durations can then be compared to the classification criteria obtained from statistical analysis, allowing for a more detailed analysis of hand-raising behavior.

### Research Questions

To understand how much work and contributions have been made in this paper, the following four research questions are proposed:

1. Can hand-raising movements be subdivided into several stages?
2. Can hand-raising recognition be achieved based on morphological methods?
3. Can a detailed analysis of hand-raising speed and amplitude levels be achieved?
4. Can existing deep learning methods directly achieve a detailed analysis of hand-raising speed and amplitude levels?

**Figure 4:**
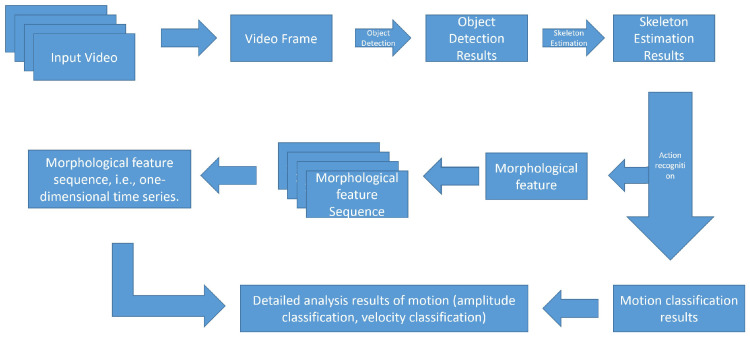
Technological roadmap.

In this article, firstly, the human skeleton result is obtained, and the hand-raising action is recognized based on the morphological method according to the skeleton estimation result. Based on this, the complete hand-raising action is divided into three stages: “beginning”, “holding”, and “ending” through morphological analysis. The classification and analysis of hand-raising speed and amplitude levels are refined, and the effects of using common deep learning methods (object detection network YOLOv4 and action recognition network ST-GCN) to achieve the above-mentioned refined analysis are compared, just as the roadmap in Figure 4.

To achieve a detailed analysis of hand-raising speed and amplitude levels in the classroom, a morphological-based method is proposed in this paper to characterize and serialize the skeleton estimation results obtained from the neural network. The time series of the signed area formed by the student’s arms and the signed angle in the entire video is obtained. The complete hand-raising action is divided into three stages: “beginning”, “holding”, and “ending”, and a detailed classification of the hand-raising speed and amplitude level is achieved.

The remainder of this paper is as follows. Section 2 introduces related work and limitations. Section 3 describes the experimental dataset and skeleton estimation method. Section 4 describes the morphological-based analysis method. Section 5 presents the experimental results. Section 6 summarizes the goals achieved by the method proposed in this paper and its superiority.

## 2. Related Work

Analyzing student hand-raising behaviors in smart classrooms is a challenge already faced by the educational science community; existing work that is closer to the approach proposed in this paper is presented first, and these include the following:

1. Student attendance in a smart classroom;
2. Detection of student hand-raising behavior in a smart classroom;
3. Recognition of student abnormal behavior;
4. Analysis of student concentration in a smart classroom.

The student attendance system based on the Internet of Things has been widely studied [23,24,25]. For example, Chang, Ching Hisang proposed an effective mechanism based on the Internet of Things architecture, called the Smart Classroom Roll Call System (SCRCS), which is installed in every classroom of the university and accumulates readings from student ID card to display the actual attendance count on an LED display screen at the beginning of each class, thus preventing students from engaging in proxy attendance activities.

The detection of students raising their hands based on machine vision has also been widely studied [26,27]. For example, Zhou, Huayi et al. [26] proposed an automatic hand-raising recognition algorithm to display hand-raising activities in a real classroom scenario. The hand-raising recognition was divided into three sub-problems—hand-raising detection, posture estimation, and matching hand-raising with students. And there have also been studies on technical details for neural network training [28], in which researchers alleviated the issue of rank disorder by training a Pareto-wise end-to-end ranking classifier to simplify the architecture search process.

The recognition of abnormal student behavior based on video analysis has also been widely studied. For example, Liu, Chang et al. [29] proposed an advanced algorithm based on spatiotemporal features of poses to recognize abnormal behavior during exams. The algorithm defines some atoms abstracted from the spatiotemporal features of key points, which can efficiently extract single-person abnormal behavior and multi-person abnormal behavior. The algorithm can distinguish various abnormal scenes such as raising hands, turning around, and passing materials.

The analysis of student concentration based on smart classrooms has also been widely studied [30,31,32,33]. For example, Chatterjee, Rajdeep et al. [33] used a real-time facial monitoring system based on deep learning to recognize the facial expressions of student and eye-opening degrees, and calculated the net learning behaviors of students by weighted averaging.

## 3. Skeleton Estimation

### 3.1. Dataset Preparation

In order to better achieve a detailed analysis of hand-raising behaviors, researchers recorded students’ hand-raising behaviors in a simulated intelligent classroom scenario, including videos of individual hand-raising and videos of multiple students raising their hands. In these videos, the hand-raising actions of students reflect different ranges and speeds, and the range and speed classifications are clear.

Specifically, the researchers divided hand-raising amplitude and speed into three categories: “big”, “medium”, and “small” for amplitude and “quick”, “medium”, and “slow” for speed. For each combination of hand-raising amplitude and speed (there were nine combinations in this experiment), a video of approximately 10 min in length was recorded, as well as a video of a student with no movements, for a total of 16 videos (10 single-person videos were used to find patterns, and 6 multi-person videos were used to verify the patterns).

Unlike many datasets used in research, the dataset in this paper was specifically recorded, so it reflects a significant change in students’ enthusiasm for raising their hands, as represented by the different ranges and speeds of hand-raising actions of students. Therefore, this is beneficial for the subsequent analysis of hand-raising amplitude and speed, as well as the analysis of hand-raising enthusiasm.

#### 3.1.1. Video Data Acquisition

The video data used in this study were recorded using a camera. Firstly, hand-raising amplitude and speed were divided into three categories each: “big”, “medium”, and “small” for amplitude, and “quick”, “medium”, and “slow” for speed. For each combination of hand-raising speed and amplitude actions (there were nine combinations in this experiment), a video of approximately 10 min in length was recorded. In a complete video, researchers raised their hands multiple times, and the video included instances of raising the left and right hands separately, but only one speed–amplitude combination appeared. These videos were processed, and morphological analysis methods and conclusions were obtained through analysis.

Meanwhile, researchers also recorded videos of multiple people raising their hands, which included not only instances of individuals raising their hands but also situations where multiple people raised their hands at the same time. These videos were also processed, and morphological analysis methods and conclusions were obtained through analysis.

#### 3.1.2. Video Data Processing

For convenience in the study, long videos were edited, resulting in short videos that sometimes contained only one instance of hand-raising, while others contained multiple instances, but all were brief and did not exceed 2 min. These short videos were input into neural networks (i.e., YOLOX and HrNet) constructed by researchers to obtain skeletal estimates. These skeletal estimates were saved in json format for subsequent research and analysis.

### 3.2. Skeleton Estimation

To achieve skeletal estimation, researchers coupled two different models, YOLOX and HrNet. For a given video, the video frames were first input into the YOLOX network to obtain the position of each person in each frame. Then, these positions were input into the HrNet network to obtain skeletal estimates for each person in each frame. For any given person, the skeleton estimation includes the coordinates of 17 skeletal key points and a confidence score. These output skeletal estimations were saved in json format, with one json file corresponding to each video.

**Figure 5:**
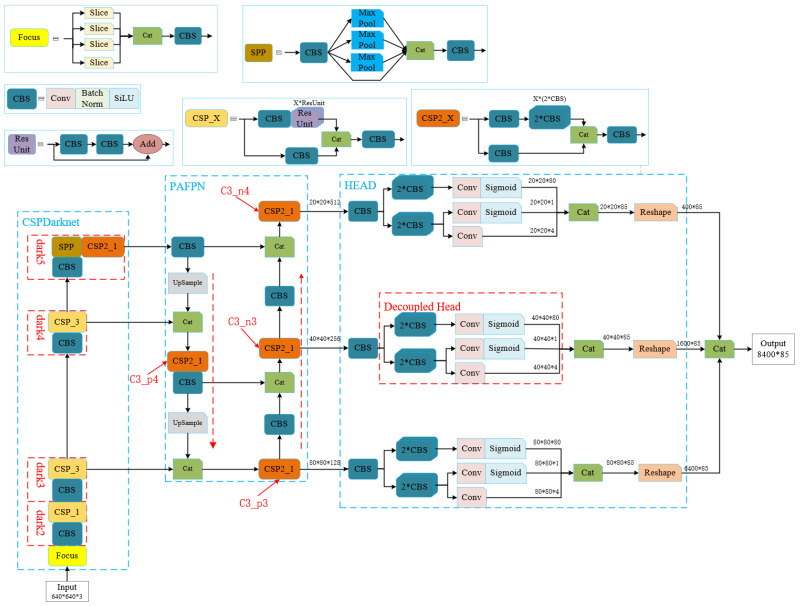
YOLOX detection network.

**Figure 6:**
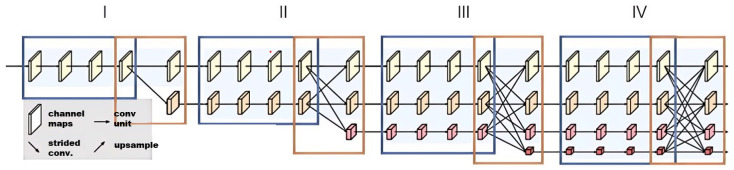
Skeleton estimation network, HRNet. In the first stage there is only one convolutional stream with the highest resolution, and a new lower resolution tributary is added after entering the next stage, and they are parallelized. For each new parallel DC, the resolution is halved and the number of channels is doubled.

For the YOLOX network, its network structure is shown in Figure 5, and consists of three parts: a backbone network, a neck network, and a prediction head. The backbone network is used to extract high-level features, the neck network is used for feature fusion, and the prediction head is used to predict the object’s class, boundary box, and other information. For the HrNet network, its network structure is shown in Figure 6, and it consists of two parts: a backbone network and a prediction head. The backbone network is used to extract high-level features, and the prediction head is used to estimate the coordinates of 17 skeletal key points. In the experiment, pre-trained detector weights for people were used.

For morphological analysis, the focus was on analyzing individual hand-raising videos, examining the characteristics of the entire hand-raising process, and conducting a detailed analysis of the different features of hand-raising speed and amplitude to obtain classification results. The proposed methodology was then validated in videos of multiple people raising their hands.

For the YOLOX network, the specific network used in this paper is YOLOX-s, with an image input size of 640 × 640, Params of 9 M, and FLOPs of 26.8 G.

In Figure 5, CSPDarknet53 is selected as the backbone network to extract high-level semantic information from the image, which solves the problem of gradient repetition during optimization. Next, path aggregation fusion (PAFPN) is used to fuse the features extracted by the backbone network. PAFPN contains a top-down path to fuse semantic information of low-resolution features and a bottom-up path to fuse spatial information of high-resolution features, which helps improve the performance of object detection. After PAFPN, three fused features are obtained, with scales of 20 × 20, 40 × 40, and 80 × 80, respectively. In the final stage, YOLOX uses three parallel and decoupled head networks to detect students, and to output bounding boxes, and confidence scores for the bounding boxes of each feature grid.

For the HrNet network, the specific network used in this paper is hrnet-w32, with an input size of 256 × 256. The model used weights trained on the MS-COCO dataset.

Skeleton estimation is a position-sensitive visual task. HrNet has achieved relatively good performance in skeleton estimation tasks, thanks to information exchange between high-resolution and low-resolution representations [22]. As shown in Figure 6, the results of the object detection network (i.e., bounding boxes) are used to crop human objects from the image and adjust them to a fixed size of 256 × 256. Then, these scaled human detection results are input into the HrNet network trained on the MS-COCO dataset [34].

The MS COCO (Microsoft Common Objects in Context) Skeleton Key Points Dataset is a widely used dataset for computer vision and machine learning research. This dataset contains over 300,000 images and more than 25,000 annotated human skeleton key points. Each human skeleton key point consists of 17 points, including the nose, neck, shoulders, elbows, wrists, hips, knees, ankles, eyes, and mouth. The coordinates of these key points can be used to identify and track human pose, as well as to analyze and understand human actions. The MS COCO Skeleton Key Points Dataset provides a valuable resource for researchers and practitioners in the field of computer vision and machine learning.

The experiment was conducted on a server equipped with two Intel(R) Xeon(R) CPU E5-2678 v3 @ 2.50 GHz processors and an Nvidia Geforce 2080Ti GPU. The operating system used was Ubuntu18.04.

In this paper, due to the fact that subsequent machine learning algorithms, such as ST-GCN, can only provide rough motion recognition, such as whether the hands are raised or not, in order to obtain more detailed motion descriptions, a morphological analysis scheme is designed, which is also the main contribution of this paper.

## 4. Morphological Analysis

This article focuses on the application of hand-raising analysis in actual smart classrooms. Based on the research presented in this article, auxiliary teaching tools (such as smart robots) in smart classrooms can score the hand-raising actions of students and select students to answer questions based on their level of enthusiasm among multiple raised hands, which is beneficial for subsequent teaching quality analysis and improvement. Therefore, morphological methods are introduced in this article to transform the results of human skeleton estimation into a time series and conduct a detailed analysis of the time series. At present, a large number of hand-raising analysis studies directly use neural networks to recognize hand-raising actions by inputting single images. For example, Gege Zhang et al. processed classroom videos in [35] and trained hand-raising action frames using box selection. This method can only recognize the action of raising hands, and since the input data for each inference involve single-frame images, it cannot conduct a detailed analysis of the time sequence, such as amplitude and speed of hand-raising actions. Additionally, this article employs skeleton estimation, which provides a more accurate analysis than directly using object detectors for hand-raising action recognition.

In the context of this study, the skeleton estimation network provides a set of data, with each detected person containing 17 points. The main focus of this study is to analyze the situation of students raising their hands in class. To achieve this goal, it is necessary to extract relevant information from the 17 points provided by the skeleton estimation network. Specifically, the analysis focuses on 6 out of the 17 points, including the left shoulder, left elbow, left wrist, right shoulder, right elbow, and right wrist. These points are crucial for analysis because they indicate the motion of the arm joints when raising hands and provide basic information about the motion.

However, understanding and analyzing these 17 skeletal sequences is not easy for a computer. Therefore, two areas are defined, namely AreaLeft and AreaRight, as well as two angles, AngleLeft and AngleRight, which form four time sequences that describe the process of raising a hand.

Selecting these four features for study was a deliberate decision. First of all, based on common sense reasoning, the action of raising hands is mainly completed by the arms and has nothing to do with the lower part of the body (i.e., legs and trunk). Therefore, the morphological analysis method introduced in this article mainly analyzes the morphological features formed by the key points on the arms. Based on a large number of experiments and analysis, two morphological features that can be used to describe the action of raising hands were discovered, namely the triangular area formed by the arms and the angle formed by the arms at the elbow. The area formed by the arms changes continuously and shows regularity as the action of raising hands proceeds. In order to obtain a more detailed description, this article studies the signed area of this region. As for the angle formed by the arms at the elbow, the most obvious observation is that when the amplitude of raising hands is the largest, the angle approaches 180°. Moreover, for raising hands with different amplitudes, there are different characteristics of the angle during the phase of holding the hand still. Therefore, this article studies two morphological features, the area formed by the arms and the angle formed by the arms at the elbow.

### 4.1. Calculation Method for Signed Area of Polygon

Firstly, the methods of computing the signed area of a polygon are introduced. And both geometric and algebraic interpretations are provided.

**Figure 7:**
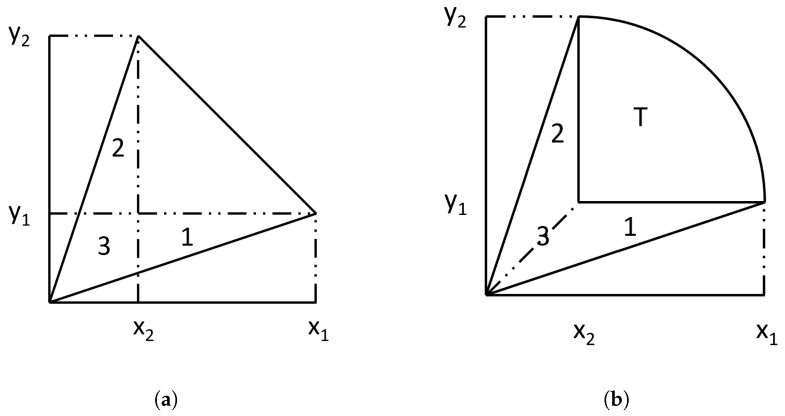
The area of Triangle 3 is equal to $\frac{1}{2}$ times the sum of the areas of Trapezoid 1 and Trapezoid 2; that is, $\frac{1}{2}\left(Area_{Trapezoid1}+Area_{Trapezoid2}\right)$. (**a**) All edges are line segments. (**b**) The line segment in the upper-right corner has been replaced by an arc.

First consider the three shapes that are most used, which are vertical strips, horizontal strips, and polar triangles. These lead to ∫f(y)dx, ∫f(x)dy, and $\frac{1}{2}\int r^{2}dr$ for the areas marked 1, 2, and 3 in the figures. As shown in Figure 7, for the convenience of calculating the area, an important assumption is that the vertex in the lower-left corner is the origin; that is, (0,0).

The area of Triangle 3 is equal to $\frac{1}{2}$ times the sum of the areas of Trapezoid 1 and Trapezoid 2; that is, $\frac{1}{2}\left(Area_{Trapezoid1}+Area_{Trapezoid2}\right)$. This conclusion is self-evident, but the proof is provided here for completeness.

For the case where all edges are line segments, the area of Trapezoid 1 is equal to $\frac{1}{2}\left(x_{1}-x_{2}\right)\left(y_{1}+y_{2}\right)$ and the area of Trapezoid 2 is equal to $\frac{1}{2}\left(x_{1}+x_{2}\right)\left(-y_{1}+y_{2}\right)$. Therefore, the sum of the areas of Trapezoid 1 and Trapezoid 2 is $\frac{1}{2}\left(x_{1}-x_{2}\right)\left(y_{1}+y_{2}\right)+\frac{1}{2}\left(x_{1}+x_{2}\right)\left(-y_{1}+y_{2}\right)$, which simplifies to $\left(x_{1}y_{2}-x_{2}y_{1}\right)$. To calculate the area of Triangle 3, the areas of the two triangles were subtracted from the area of the entire region, which is $\left(x_{2}y_{2}\right)+\frac{1}{2}\left(x_{1}-x_{2}\right)y_{1}-\frac{1}{2}\left(x_{1}-x_{2}\right)\left(y_{1}+y_{2}\right)-\frac{1}{2}x_{1}y_{1}-\frac{1}{2}x_{2}y_{2}$. This equation is equal to $\frac{1}{2}\left(x_{1}y_{2}-x_{2}y_{1}\right)$, which proves that the area of Triangle 3 is equal to $\frac{1}{2}\left(Area_{Trapezoid1}+Area_{Trapezoid2}\right)$.

Similarly, for the case where there are arcs in Figure 7, let *T* be the area of the small triangle enclosed by the arc. Then, the area of Trapezoid 1 is equal to $T+\left(x_{1}-x_{2}\right)y_{1}$, and the area of Trapezoid 2 is equal to $T+x_{2}\left(y_{2}-y_{1}\right)$. The area of region 3 can also be expressed as $T+\frac{1}{2}\left(x_{1}-x_{2}\right)y_{1}+\frac{1}{2}x_{2}\left(y_{2}-y_{1}\right)$. After simplification, it can be seen that the area of Triangle 3 is still equal to $\frac{1}{2}\left(Area_{Trapezoid1}+Area_{Trapezoid2}\right)$. The conclusion still holds.

Therefore, for a triangle with vertices $A(x_{1},y_{1})$, $B(x_{2},y_{2})$, and C(0,0), its area can be calculated using the formula $\frac{1}{2}\left(x_{1}y_{2}-x_{2}y_{1}\right)$.

Based on this, the question of how to calculate the signed area of any triangle can be answered.

**Figure 8:**
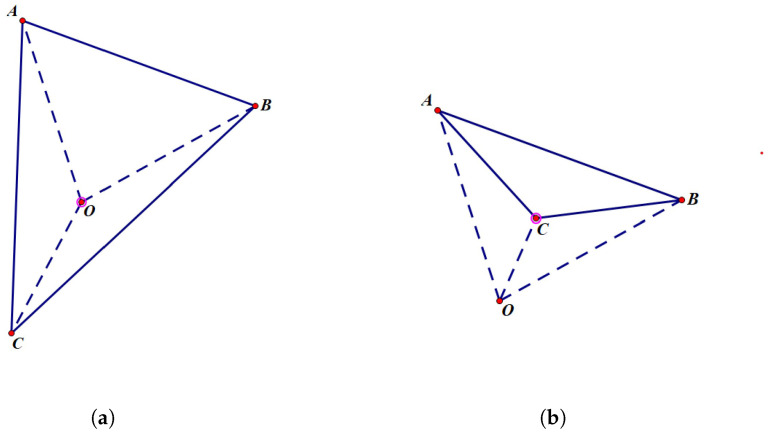
Any polygon can be decomposed into the sum of the signed areas (with clockwise orientation still being positive) of multiple triangles. (**a**) Origin is inside triangle ABC. (**b**) Origin is outside the triangle ABC.

First, for any triangle, in order to calculate its signed area (with the convention that clockwise orientation is positive), it can be decomposed into the sum of the signed areas (with clockwise orientation still being positive) of multiple triangles. Here, *O* is taken as the origin, as shown in Figure 8. If the origin is inside triangle ABC, as is shown in the left subfigure in Figure 8, the triangle ABC is divided into three sub-triangles by the dashed lines, namely AOB, AOC, and BOC. The signed area of triangle ABC is equal to the sum of the signed areas of these three sub-triangles; otherwise, if the origin is outside triangle ABC, as is shown in the right subfigure in Figure 8, the triangle ABC is divided into three sub-triangles by the dashed lines, namely AOB, AOC, and BOC. The signed area of triangle ABC is equal to the signed area of the large triangle AOB minus the signed areas of the remaining two small triangles AOC and BOC.

**Figure 9:**
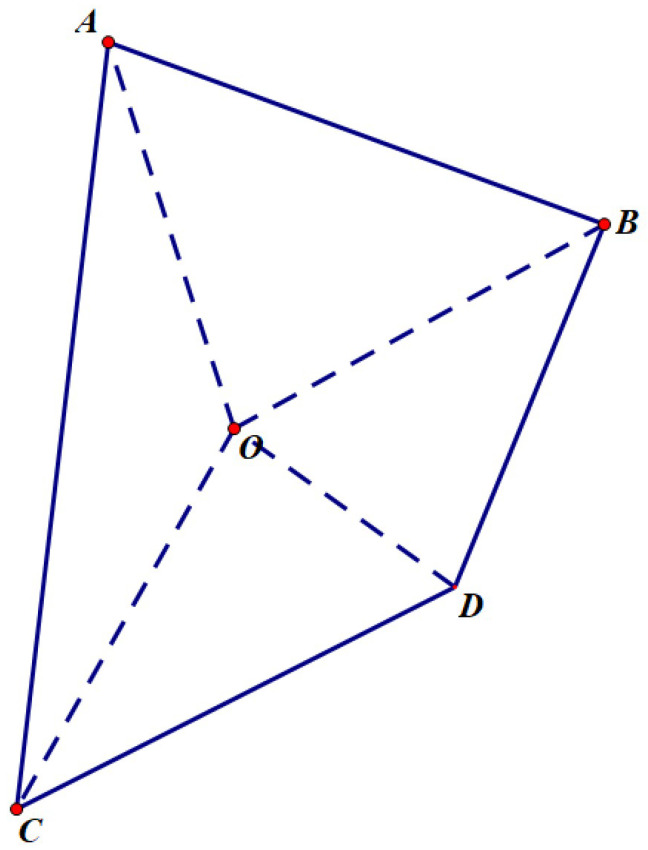
Any polygon can also be decomposed into the sum of the signed areas (with clockwise orientation still being positive) of multiple triangles.

Similarly, when the polygon has more edges than a triangle, in order to calculate its signed area (with the convention that clockwise orientation is positive), it can also be decomposed into the sum of the signed areas (with clockwise orientation still being positive) of multiple triangles. Here, *O* is taken as the origin, as shown in Figure 9.

Therefore, for any polygon, its signed area can be quickly calculated using the Formula (2).

Define $P_{1},\;P_{2},\;\cdots \;,P_{N}$ as ordered vertices of a polygon, and let ${\left(x_{n},y_{n}\right)}^{T}$ be the coordinate of the vertex $P_{n}$. Then, the (signed) area of the polygon $P_{1}P_{2}\cdots P_{N}$ can be denoted by $S(P_{1}P_{2}\cdots P_{N})$, which can then be calculated (note that the index is *circulated*. That is, if n+1>N, then $P_{n+1}=P_{1}$; moreover, if n−1<1, then $P_{n-1}=P_{N}$).

(1) $$\begin{array}{cc} S & \left(P_{1}P_{2}\cdots P_{N}\right)=\frac{1}{2}\sum_{n=1}^{N}x_{n}\left(y_{n+1}-y_{n-1}\right) \end{array}$$

(2) $$\begin{array}{cc} \; & =\frac{1}{2}\left[x_{1}\left(y_{2}-y_{N}\right)+x_{2}\left(y_{3}-y_{1}\right)+\cdots +x_{N-1}\left(y_{N}-y_{N-2}\right)+x_{N-1}\left(y_{1}-y_{N-1}\right)\right] \end{array}$$

In this study, for each student, three areas were mainly calculated, namely, the area formed by the left arm, the area formed by the right arm, and the area of the student’s body trunk. Calculating the area formed by the arms involved transforming the time series of skeleton estimation results into a one-dimensional time series and analyzing it using signal processing concepts. Calculating the area of the body trunk was primarily aimed at improving the robustness of the method. For example, this calculation helped offset the influence of varying distances between the cameras and different students in various situations. It ensured that the information on the amplitude of hand-raising from students located in different directions in the classroom could be compared equally.

To analyze the hand-raising motion of students in class, it is necessary to focus on the arms that may represent the hand-raising motion, as shown in Figure 1b. An arm can provide a polygon. A student has two arms, so for each target, it is necessary to consider both the left and right arms. For example, the first area is surrounded by the left wrist, left elbow, and left shoulder, and the second area is surrounded by the right wrist, right elbow, and right shoulder. The areas of these two regions are AreaLeft and AreaRight, respectively. To describe these two areas, the signed area of the polygon needs to be calculated. The calculation method for the signed area is as follows:

In Figure 1b, the green dots represent the key points on the left arm, the blue dots represent the key points on the right arm, and the black lines connect the area enclosed by the left arm, while the red lines connect the area enclosed by the right arm.

After careful observation of the process of a single person raising their hand, several findings can be obtained:

1. **First observation:** Regardless of how the student moves, the order of the four key points that describe their body area remains fixed. These four key points are the left shoulder, right shoulder, right hip, and left hip.
2. **Second observation:** The distance of different students from the camera can lead to variations in the apparent size of the students’ bodies in the video.

These two observations indicate that using the signed area of a polygon to describe the student’s motion is reasonable, but the original polygon area needs to be standardized by the body area. With the coordinates of the key points, the signed area of the polygon can be calculated. At the same time, it is also necessary to ensure that the fixed point order used to calculate the signed area of the region must remain consistent.

The problem with calculating the area of a polygon has been well-studied and these signed areas can now be effectively calculated.

The body area, i.e., the area enclosed by the four key points of the left shoulder, right shoulder, right hip, and left hip, can be calculated using the formula in Equation (2).

For the area enclosed by the three key points of the arm, elbow, and wrist, taking the left arm as an example, the formula in Equation (2) is used for calculation, as shown in Figure 1b.

Two angles are also defined as AngLeft and AngRight, to form two time series, describing the process of raising hands.

### 4.2. Calculation of Angles

In this study, for each student, two angles are primarily calculated: the angle formed by the left arm at the elbow and the angle formed by the right arm at the elbow. This approach involves transforming the time series of skeleton estimation results into a one-dimensional time series and analyzing it using signal processing concepts.

**Figure 10:**
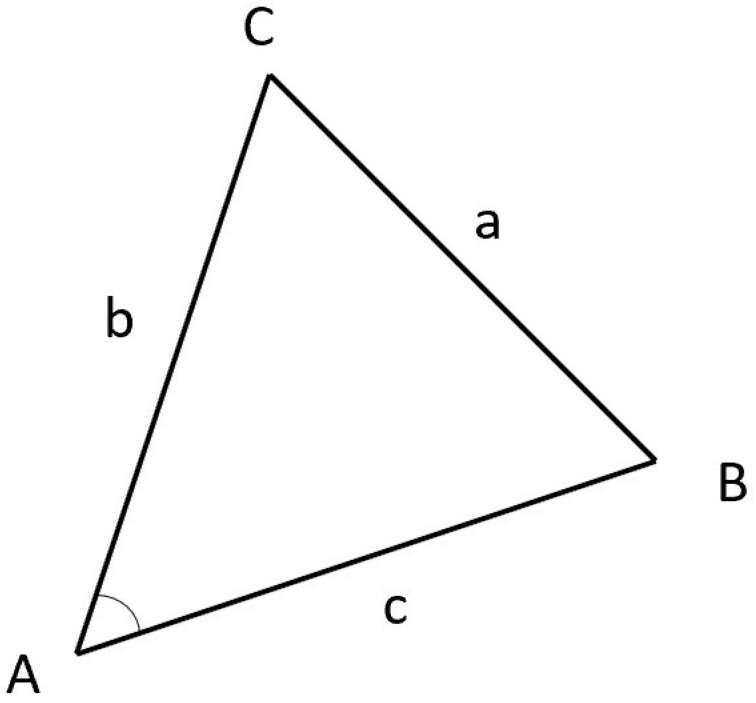
The cosine law allows the calculation of any angle.

The cosine law states that—for a triangle composed of three vertices—the angle corresponding to each vertex can be calculated given the lengths of the three sides, as shown in Figure 10. In Figure 10, *A*, *B*, and *C* represent the three vertices; *a*, *b*, and *c* represent the three sides, and *A*, *B*, and *C* represent the three angles. By using the cosine law, the angles of the three vertices can be calculated.

Based on this, the corresponding angle values can be calculated using the inverse cosine function, and the time series formed by these angle values can be analyzed in subsequent steps.

Through the above process, the results of human skeleton estimation in a single image can be represented by morphological features, and in a sequence of multiple images (such as a video), a time series of geometric parameters can be formed. The time series obtained through the above process can effectively describe the motion information of students raising their hands. By utilizing this information, more advanced features can be extracted.

## 5. Analysis of Feature Sequences

After obtaining the above one-dimensional time series, detailed analysis and scoring of hand-raising behavior can be achieved through further analysis. In the smart classroom scenario, the designated areas and angles that constitute the one-dimensional time series can describe the amplitude and speed of hand-raising, and the process of student hand-raising can be analyzed by analyzing these time series. In this section, the process of a single student raising their hand is first analyzed.

It is worth noting that—in order to enhance the robustness, in addition to using the area of the body trunk to normalize the area formed by the arms—this study also considers the special situations that may exist in the angle sequence. For example, when the positions of two key points in the triangular area formed by the arms are the same, the calculated angle is 0. This situation is impossible in practical applications. Therefore, this study sets the angle value of this situation to the angle value of the previous frame, which can ensure the continuity of the angle sequence.

At the same time, based on the consideration of the reliability of skeleton estimation results, and referring to the previous research on the credibility of skeleton estimation results by scholars, this method is used for similar processing. That is, when the skeleton estimation results of a certain frame are not credible, the area value of that frame is set to the area value of the previous frame, which can ensure the continuity of the area sequence.

**Figure 11:**
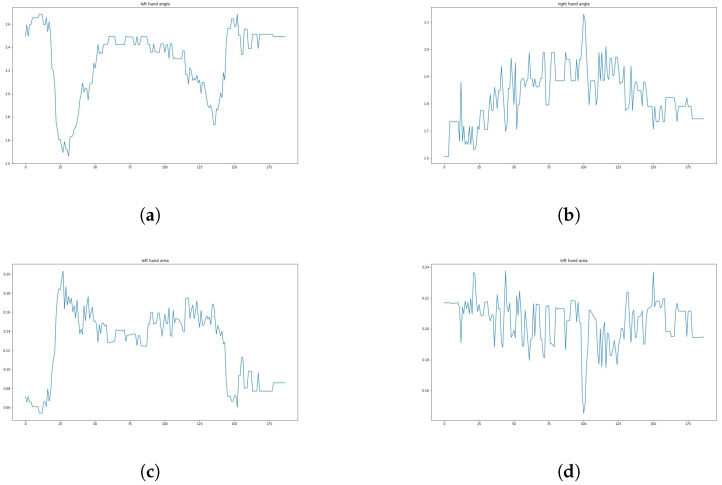
Visualization results of the feature sequence studied in this paper. (**a**) The angle sequence of the left elbow. (**b**) The angle sequence of the right elbow. (**c**) The left arm area sequence. (**d**) The right arm area sequence.

**Figure 12:**
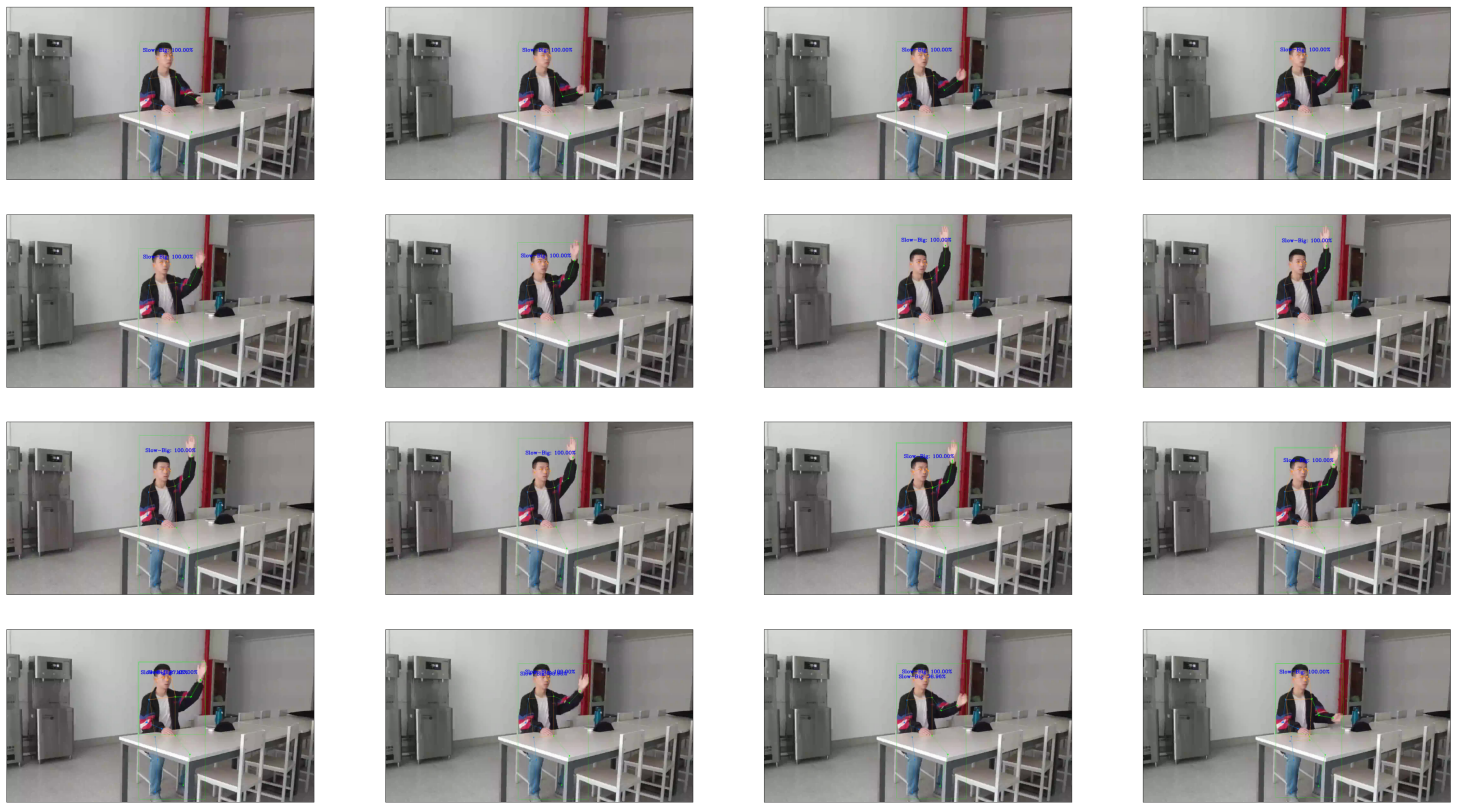
A time series of image frames from a sample video.

Regarding a student’s arm—its motion can be described by a time series of the triangular area and angle. Therefore, the area calculated in the previous section can be used to describe the student’s hand-raising motion. After this processing, the student’s motion is transformed into four one-dimensional time series (normalized by body area), as shown in Figure 11, which shows an example of these four time series in a video (see Figure 12). The video contains 189 frames, recorded at a frame rate of 25 frames per second, and lasts 7.56 s. Therefore, the length of the time series is 189.

As shown in Figure 11, the process of raising hands is clearly reflected in these four one-dimensional signals (since in most cases, a student raises only one hand, the sequence of regions and angles describing one arm will show a significant regularity, while the sequence of the other pair of regions and angles may exhibit a stable state). The horizontal axis represents the frame numbers corresponding to the feature quantities in the video, and the vertical axis represents the size of the feature quantity for each frame (where the area has been normalized). In this video, the student raised their left hand but did not raise their right hand. This sub-figure shows the angle sequence at the student’s left elbow in the example video. Compared with the angle sequence at the student’s right elbow (i.e., the sub-figure on the right), it can be clearly seen that the student only raised their left hand. By observing this sub-figure, the whole process of raising one’s hand can be analyzed: first, the “start” phase from frame 18 to frame 56, then the “maintain” phase from frame 57 to frame 114. Finally, the “end” phase from frame 115 to frame 168. It can be seen clearly that the student only raises his left hand without raising his right hand.

Through morphological analysis, the time sequence of the student’s skeleton is simplified and transformed into one-dimensional time sequences of the arm region area and arm angle, called feature sequences in this paper. Based on the feature sequences, the student’s hand-raising action can be analyzed in more detail.

In this study, morphological analysis was performed on the data obtained from the skeleton estimation network to study a student’s hand-raising action. The aim was to extract arm angle and area information from the coordinate information of key points. After the analysis, a very unique feature was found, which divided the entire hand-raising process into three stages.

The first stage, called the “start” stage, is characterized by rapid changes in arm angle and area when the student’s hand is raised from the initial position. In this stage, the student’s hand moves from a stationary position to a raised position.

The second stage, called the “hold” stage, occurs when the student’s hand is held high and the arm angle and area remain relatively stable. During this stage, the student’s hand is raised and there is no significant change in the numerical value of the arm angle area.

The last stage, called the “end” stage, is characterized by rapid changes in the arm angle and area when the student’s hand returns from a raised position to the initial position. In this stage, the student’s hand moves from a raised position to a resting position.

**Figure 13:**
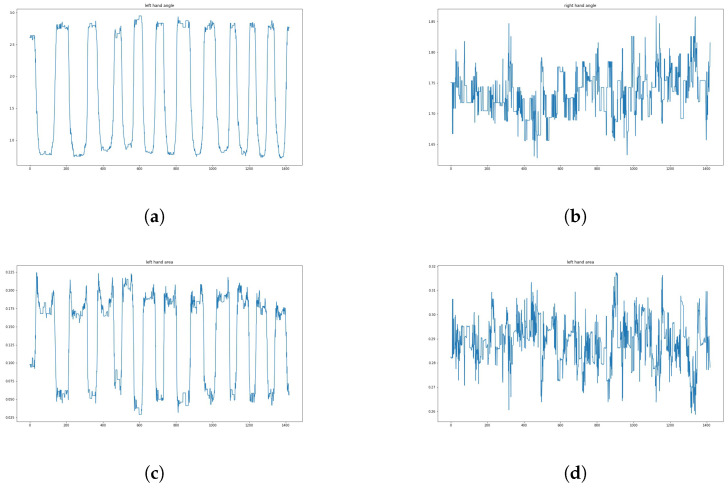
Visualization results of the feature sequence studied in this article. (**a**) Angle sequence of left elbow. (**b**) Angle sequence of the right elbow. (**c**) Area sequence of left elbow. (**d**) Area sequence of the right elbow.

From Figure 13, it can be observed that the morphological features exhibit periodicity during continuous hand-raising. The horizontal axis represents the frame number corresponding to the feature in the video, and the vertical axis represents the magnitude of the feature in the corresponding frame (where the area has been normalized). In this video, a student repeatedly raises their left hand while keeping their right hand relatively still. Compared with the angle sequence at the student’s right elbow (i.e., the sub-figure on the right), it can be clearly seen that the student only raised their left hand. By observing this sub-figure, the periodicity can be clearly observed. From the area enclosed by the student’s left arm in the same hand-raising video, it can be clearly seen that the student only raised their left hand.

A typical complete hand-raising process of a student can be represented by these three stages, namely “start”, “hold”, and “end”. The results of this analysis are shown in Figure 11.

A question worth considering is how to define the beginning and end of the entire hand-raising process. To solve this problem, existing deep learning-based hand-raising detection algorithms can be combined. That is, the recognition of hand-raising action by the hand-raising detection algorithm is used as the beginning of the entire hand-raising process, and the end of the entire hand-raising process is defined as when the hand-raising detection algorithm does not detect hand-raising action within ten consecutive frames. In this way, the beginning and end of the entire hand-raising process can be determined by the recognition results of the hand-raising detection algorithm.

Within the process confirmed as the hand-raising stage, by analyzing the sequence obtained through the processing of skeleton estimation results using the morphological analysis method, detailed information on the speed and amplitude of student hand-raising can be obtained. This work is unique to this study.

The speed of a student’s hand-raising can be used as an effective indicator to describe the hand-raising action. Taking the “start” stage as an example, the time elapsed between the start and end of the stage can describe the speed of a student’s raising hand. That is, the longer the duration of stage one, the slower the raising hand, and the shorter the duration of stage one, the faster the raising hand.

**Figure 14:**
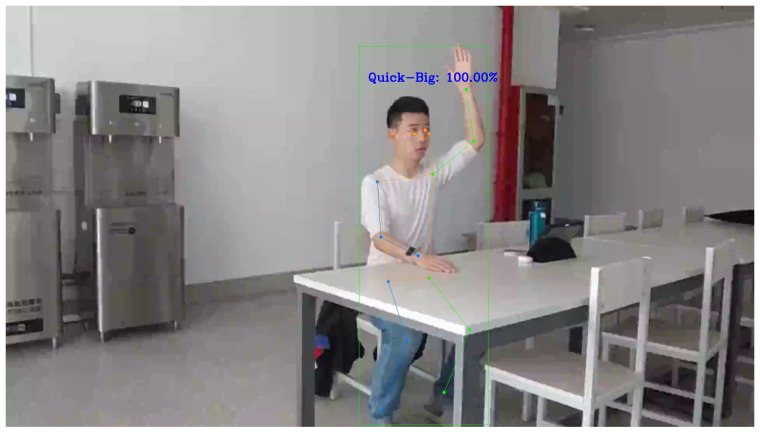
When the hand-raising amplitude is the largest, the student’s arm is close to upright.

Similarly, the size of the hand-raising amplitude is also a significant feature that can be used to describe the hand-raising action, analyzed from the arm area change and angle change. For stage one, i.e., the “start” stage, looking at the arm area change, the larger the area change in stage one, the greater the hand-raising amplitude, and the smaller the area change in stage one, the smaller the hand-raising amplitude. Looking at the arm angle change, since the hand-raising amplitude is the largest when the student’s arm is close to upright, as shown in Figure 14, the angle is close to 180 degrees when stage one ends, indicating the maximum hand-raising amplitude.

In addition, an analysis of the angle sequences of the typical small, medium, and large amplitudes of raising hands revealed that only for large amplitudes does the angle change significantly in a short period of time: as shown in Figure 14, the angle first decreases (the arm shrinks from a flat position, causing the angle to decrease rapidly), and then increases (after the arm shrinks, because the amplitude is large, the arm will visibly expand, causing the angle to increase rapidly), while no such feature is observed in the angle sequences of small and medium amplitudes.

Based on the above ideas, the student’s hand-raising action can be divided into nine categories when analyzing real situations, including “quick speed—big amplitude”, “quick speed—medium amplitude”, “quick speed—small amplitude”, “medium speed—big amplitude”, “medium speed—medium amplitude”, “medium speed—small amplitude”, “slow speed—big amplitude”, “slow speed—medium amplitude”, and “slow speed—small amplitude”. Refer to Table 1.

For the analysis of a single person’s single hand-raising scene, taking the video corresponding to Figure 11 and Figure 12 for example, the starting points of stages one, two, and three can be clearly identified. Next, a detailed analysis is conducted: the entire video contains 189 frames, and 25 frames are recorded per second. Stage one lasts for 23 frames or 0.92 s; stage two lasts for 48 frames or 1.92 s; and stage three lasts for 56 frames or 2.24 s. If we look at Figure 12, it shows a time series of image frames from a sample video, which has 187 frames. At a frequency of taking one image every five frames starting from the first frame, 36 images are captured until the 181st frame, showing a complete hand-raising process. The figure should be viewed from left to right. The three stages of hand-raising can be clearly observed: the first column from the first to the fourth image is before the student raises their hand. The fifth image of the first column to the fourth image of the second column denotes the “beginning” stage of the student’s hand-raising, during which the student’s hand is raised. The fifth image of the second column to the first image of the fifth column denotes the “holding” stage of the student’s hand-raising, during which the student’s hand is held up without significant changes in amplitude or speed. The second image of the fifth column to the first image of the sixth column denotes the “ending” stage of the student’s hand-raising, during which the student lowers their raised hand. The second image of the sixth column to the last image is after the student raises their hand.

Obviously, the detailed analysis mentioned above cannot be directly extracted from the skeleton information.

## 6. Experimental Results

Despite numerous studies on hand-raising in classrooms, many have only conducted basic hand recognition without detailed analysis. In classroom settings, such as those involving robotic teaching, a deeper understanding of hand-raising is crucial for improving student instruction. The morphological method proposed in this paper can precisely classify the speed and amplitude of student hand-raising. This allows teaching robots to rank different hand-raising behaviors by their level of activity, forming the basis for interaction between intelligent robots, systems, and students in smart classrooms. Based on the morphological method, experiments were conducted on single and multiple hand-raising videos with varying speeds and amplitudes.

After obtaining key information on the speed and amplitude of students raising their hands, this paper enables a detailed analysis of each student’s enthusiasm. The analysis simulates a classroom environment where multiple students are raising their hands simultaneously. For each student, a sequence of six key points for the left and right arms is obtained. By analyzing the signal sequences of each student individually, it is possible to determine whether a student is currently raising their hand. Furthermore, the student’s enthusiasm in answering questions can be inferred from their hand-raising speed, which is assessed using the method proposed above.

The videos used in the experiment are mainly divided into two categories: single and multiple hand-raising videos. The videos recorded include 10 categories: “no action”, “quick speed—big amplitude”, “quick speed—medium amplitude”, “quick speed—small amplitude”, “medium speed—big amplitude”, “medium speed—medium amplitude”, “medium speed—small amplitude”, “slow speed—big amplitude”, “slow speed—medium amplitude”, and “slow speed—small amplitude”. And the dataset is created deliberately by researchers. For each category, first record one-time hand-raising movements, then record videos of constant hand-raising. These videos are then divided into several parts, according to video length, with each segment lasting no more than 2 min. This division facilitates neural network training and subsequent analysis. These videos were recognized as “raising hands” by existing action recognition algorithms. However, the morphology-based method proposed in this paper can provide a more detailed description of the “raising hands” behavior.

Table 2 summarizes detailed analysis results for some videos. These videos were roughly recognized as “raising hands” by mainstream action recognition networks such as ST-GCN. However, the morphology-based hand-raising analysis method proposed in this paper not only successfully detects the hand-raising action and divides the complete hand-raising process into three stages, but also enables detailed analysis of different hand-raising amplitudes and sizes. The experimental results show that this method can effectively detect hand-raising actions and provide a more detailed description of raising hands.

**Figure 15:**
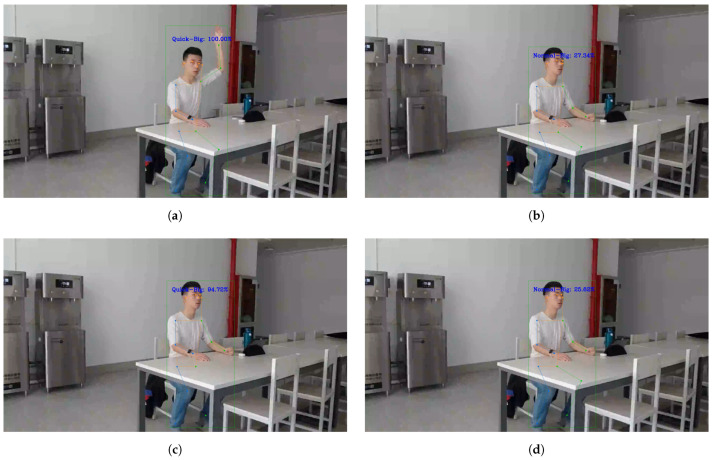
Neural network result not stable. (**a**) Neural network detects. (**b**) Neural network result jumps to “medium speed”. (**c**) Neural network result jumps back to “quick speed”. (**d**) It then jumps to “medium speed” again.

For the method of neural networks, researchers usually use it to obtain a rough classification of actions, as shown in Table 2 and Table 3. In this study, the same network structure, ST-GCN, was used for recognizing detailed sub-actions, but the experimental results were very poor. In Figure 15, for the same video, the student’s hand-raising action was described in detail as “fast speed—large amplitude”, but in the adjacent frames when the student finished the hand-raising action, the neural network recognition result jumped and the speed was recognized as “medium speed”.

The morphology-based analysis method proposed in this paper can provide a more detailed and meticulous description of the hand-raising action. It not only recognizes the hand-raising action based on the results of skeleton estimation but also divides the hand-raising process into three stages: “start”, “maintain”, and “end”. By analyzing the number of frames for each stage (which represents the duration of each stage) and the size of the hand-raising amplitude, the hand-raising action of a single person can be divided into nine categories, each representing different levels of student enthusiasm. Therefore, in a scenario where multiple students are raising their hands simultaneously, the enthusiasm levels of multiple students can be compared, which is of great significance for the implementation of a smart classroom.

There may be a situation where the speed of the “start” stage of hand-raising for two students is not significantly different. In this case, the enthusiasm of the two students can be compared by analyzing the duration of the “maintain” stage of hand-raising.

## 7. Conclusions and Future Work

The focus of this article is not on improving the existing hand-raising recognition methods in terms of accuracy, but on proposing a method based on morphology and time series analysis to answer the question of “whether it is possible to rank the different hand-raising behaviors of different students”. This is extremely important for future smart classroom scenarios with intelligent systems as teaching assistants. In general, by using the morphological method, the student’s skeleton key point information is extracted into a time series of morphological features, and the classification of hand-raising speed and amplitude is achieved through time series analysis, thereby ranking the different hand-raising behaviors of different students.

In this study, a new morphology-based method was proposed to analyze video recordings in video surveillance systems, achieving detection of hand-raising situations and detailed classification of hand-raising speed and amplitude in a simulated classroom environment. This method converts surveillance video into discrete signals representing the area and angle of the student’s arm, and identifies changes in the signal to analyze the duration of the “start”, “hold”, and “end” phases, effectively distinguishing the enthusiasm of hand-raising. While hand-raising detection has been well-studied, detecting hand-raising alone is not enough. Subsequent teaching (such as selecting a more suitable student from among many raised hands to answer a teacher’s question) requires a detailed analysis of hand-raising speed. Therefore, the work proposed in this paper is of great importance.

The method proposed in this paper has some limitations. The first is relatively weak scalability. The starting point of this study is to obtain a more detailed action analysis than the general neural network results, based on the student’s skeleton information and temporal analysis. Although it achieves a fine-grained analysis of the specific behavior of student hand-raising, this is based on manual feature extraction. In the future, research could explore automatic feature extraction to enhance this approach. Another limitation is the high computational demand. The algorithm uses two neural networks, YOLOX and HRNet, which may face computational challenges in scenarios like distributed teaching aids. However, this issue can be addressed by utilizing centralized computing centers for video analysis.

This not only helps teachers select more enthusiastic students to answer questions in the classroom but also provides the possibility of further analysis for subsequent research, such as calculating student participation and enthusiasm in the classroom, which was not covered in previous studies.

In future research, since neural networks do not excel at the detailed classification of hand-raising speed and amplitude, a method combining action recognition networks (such as ST-GCN) with morphological analysis can be used to achieve better analysis. Based on this accurate and detailed analysis of hand-raising behaviors, many innovations can be proposed in the field of intelligent classrooms. For example, teaching aids such as intelligent robots could sense and record students’ enthusiasm for raising their hands in the classroom, and automatically assess the current learning status of all students to select the most appropriate respondents to questions. This could significantly enhance the efficiency of intelligent classrooms. Additionally, this approach could improve future student learning profiles, which would intelligently summarize students’ historical and current learning statuses and compare them with other students and baselines, to achieve personalized and intelligent training for students.

## Figures and Tables

**Table 1:** Divide the student’s hand-raising actions into nine categories.

Detailed Analysis Results	Detailed Speed Analysis	Detailed Amplitude Analysis
Quick speed—Big amplitude	Quick speed	Big amplitude
Quick speed—Medium amplitude	Quick speed	Medium amplitude
Quick speed—Small amplitude	Quick speed	Small amplitude
Medium speed—Big amplitude	Medium speed	Big amplitude
Medium speed—Medium amplitude	Medium speed	Medium amplitude
Medium speed—Small amplitude	Medium speed	Small amplitude
Slow speed—Big amplitude	Slow speed	Big amplitude
Slow speed—Medium amplitude	Slow speed	Medium amplitude
Slow speed—Small amplitude	Slow speed	Small amplitude

**Table 2:** Further analysis of the single-person video.

Video Name	Manual Annotation	Morphological Results	Neural Network Results
Single1	Left Small-Quick	Left Small-Quick	HandRaising
Single2	Left Medium-Quick	Left Medium-Quick	HandRaising
Single3	Left Big-Quick	Left Big-Quick	HandRaising
Single4	Left Small-Medium	Left Small-Medium	HandRaising
Single5	Left Medium-Medium	Left Medium-Medium	HandRaising
Single6	Left Big-Medium	Left Big-Medium	HandRaising
Single7	Left Small-Slow	Left Small-Slow	HandRaising
Single8	Left Medium-Slow	Left Medium-Slow	HandRaising
Single9	Left Big-Slow	Left Big-Slow	HandRaising

**Table 3:** Further analysis of the dual-person video, considering only the “start” stage time for hand-raising.

Video Name	Person1	Frames	Person2	Frames	Neural Network Result
two1	Quick	7	Medium	24	HandRaising
two2	Medium	12	Medium	15	HandRaising
two3	Medium	16	Medium	23	HandRaising
two4	Quick	5	Medium	19	HandRaising
two5	Medium	24	Slow	36	HandRaising
two6	Slow	38	Medium	16	HandRaising

## Data Availability

Because of the privacy of the video shooting, the experimental data are not published without request.
